# Supplementary material for: Macrophage adaptation to hypoxia in the tuberculous granuloma potentiates mycobacterium-induced mitochondrial damage and granuloma necrosis
Source: bioRxiv. 2026 Feb 7:2026.02.06.702658. Preprint. [Version 1] doi: 10.64898/2026.02.06.702658 (PMC12893048; doi:10.64898/2026.02.06.702658)

bioRxiv preprint doi: https://doi.org/10.64898/2026.02.06.702658; this version posted February 7, 2026. The copyright holder for this preprint (which was not certified by peer review) is the author/funder, who has granted bioRxiv a license to display the preprint in perpetuity. It is made available under aCC-BY 4.0 International license.

Figure S1. *vhl* mutant animals have increased HIF-1 activity, related to Figure 3.

(A) Expression of HIF target gene transcripts in uninfected *vhl*$^{sa40757/sa40757}$ and phenotypically WT siblings (*vhl*$^{+/+}$ and *vhl*$^{+/-}$) 3dpf, measured by qRT-PCR and normalized to wild-type values. (B and C) Confocal micrographs of the (B) tail or (C) brain vasculature of *vhl*$^{sa40757/sa40757}$ and phenotypically WT sibling, 5dpf. Arrowheads indicate increased blood vessel abundance. Scale bar, 100 μm.

bioRxiv preprint doi: https://doi.org/10.64898/2026.02.06.702658; this version posted February 7, 2026. The copyright holder for this preprint (which was not certified by peer review) is the author/funder, who has granted bioRxiv a license to display the preprint in perpetuity. It is made available under aCC-BY 4.0 International license.

Figure S2. HIF-1 stabilization accelerates Mm-induced macrophage cell death without altering intracellular Mm burdens, related to Figure 3.

Tg(mpeg1;YFP) larvae were infected intravenously with tdTomato-expressing Mm at 2 dpf and treated with Roxadustat (60 μM) or 0.5% DMSO immediately after infection. 5 dpf (3 dpi) animals were imaged by timelapse microscopy for 4 hours. Dying macrophages were identified by rapid loss of YFP fluorescence accompanied by cellular fragmentation.

(A) Percent of infected macrophages dying per field.

(B) Intracellular Mm burdens per macrophage in the first movie frame.

(C) Intracellular Mm burdens of dying macrophages at the time of cell death.

Bars and horizontal lines indicate mean values. Symbols represent individual (A) animals or (B and (C) macrophages. (B and C) Statistical significance was determined with a two-tailed, unpaired t-test with Welch's correction (ns, $p > 0.05$).

Figure S1

bioRxiv preprint doi: https://doi.org/10.64898/2026.02.06.702658; this version posted February 7, 2026. The copyright holder for this preprint (which was not certified by peer review) is the author/funder, who has granted bioRxiv a license to display the preprint in perpetuity. It is made available under aCC-BY 4.0 International license.

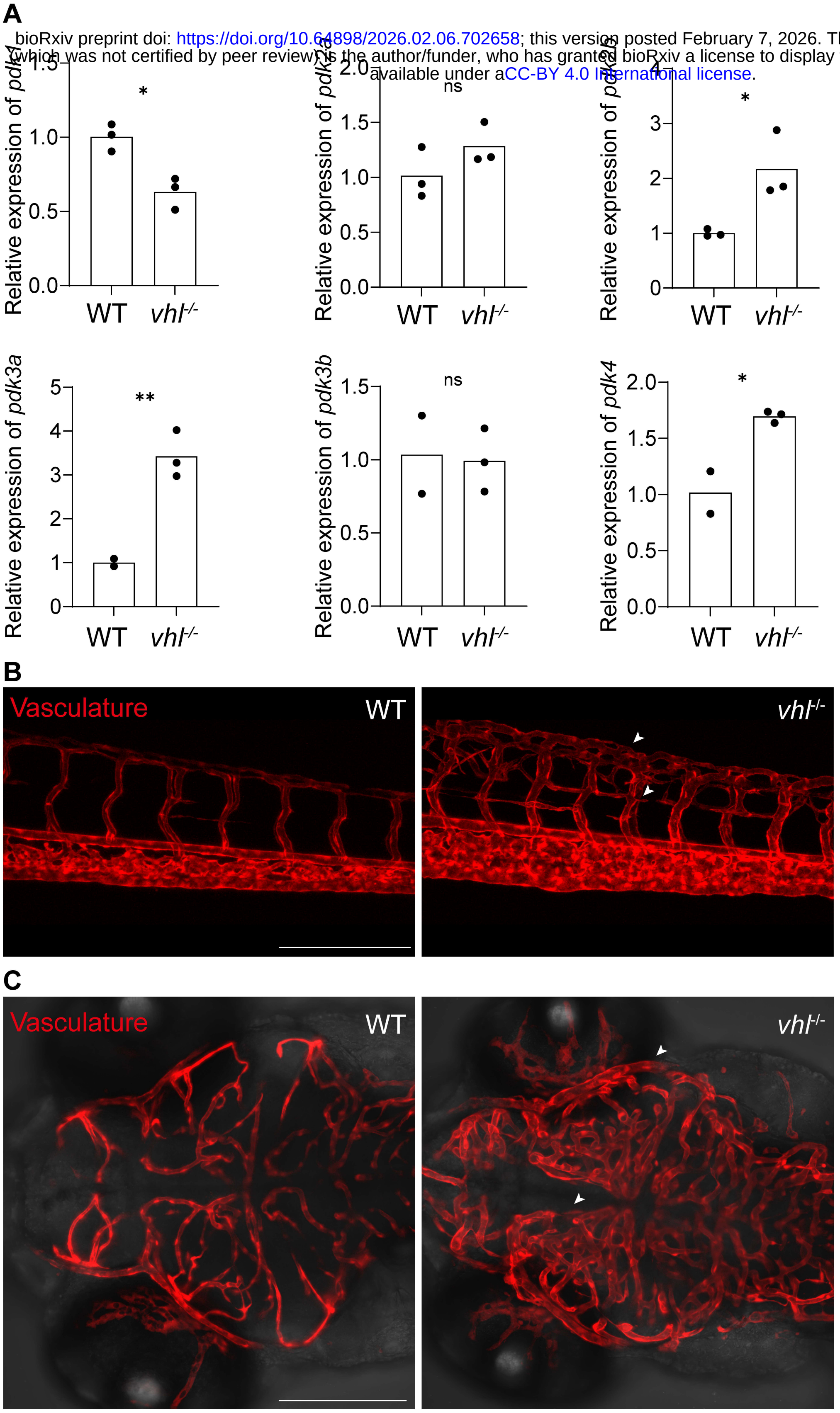

Figure S2

bioRxiv preprint doi: https://doi.org/10.64898/2026.02.06.702658; this version posted February 7, 2026. The copyright holder for this preprint (which was not certified by peer review) is the author/funder, who has granted bioRxiv a license to display the preprint in perpetuity. It is made available under aCC-BY 4.0 International license.

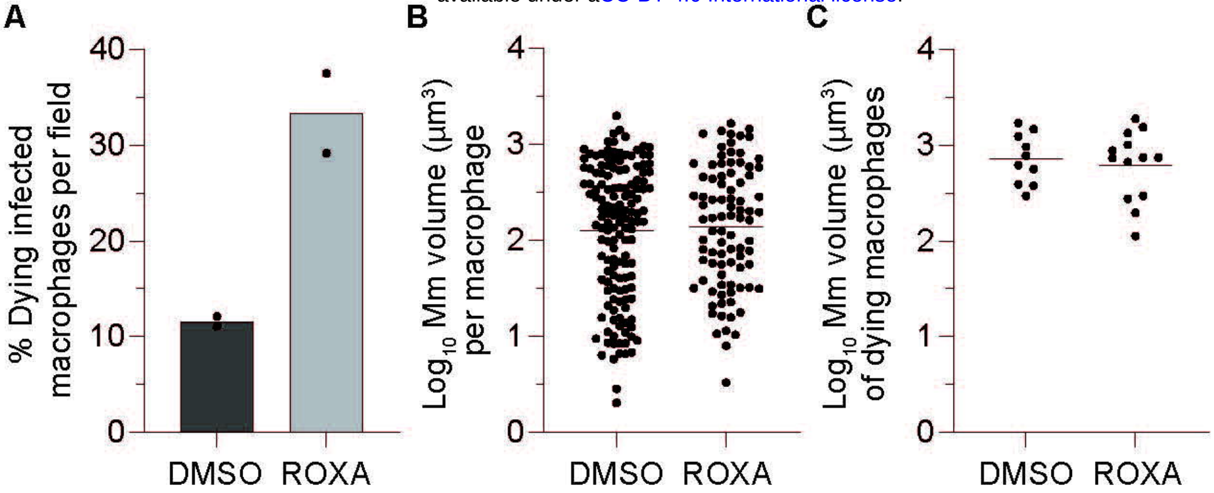

Supplement: 5 [file NIHPP2026.02.06.702658v1-supplement-5.pdf]
